# High-Aspect Ratio *β*-Ga_2_O_3_ Nanorods via Hydrothermal Synthesis

**DOI:** 10.3390/nano8080594

**Published:** 2018-08-05

**Authors:** Hyun Jeong Bae, Tae Hee Yoo, Youngbin Yoon, In Gyu Lee, Jong Pil Kim, Byung Jin Cho, Wan Sik Hwang

**Affiliations:** 1 Department of Materials Engineering, Korea Aerospace University, Goyang 10540, Korea; baehj83@gmail.com (H.J.B.); kauyootaehee@gmail.com (T.H.Y.); ybyoon93@gmail.com (Y.Y.); leeig@kau.ac.kr (I.G.L.); 2 Division of Analysis & Research, Korea Basic Science Institute, Busan 46742, Korea; jpkim@kbsi.re.kr; 3 School of Electrical Engineering, KAIST, Daejeon 34141, Korea; elebjcho81@kaist.ac.kr

**Keywords:** low dimensional structures, hydrothermal crystal growth, nanomaterials, Ga_2_O_3_, nanorods

## Abstract

High-aspect ratio *β*-Ga_2_O_3_ nanorods consisting of prism-like crystals were formed using gallium oxyhydroxide and ammonia hydroxide via a hydrothermal synthesis followed by the subsequent calcination process. The formation of high-aspect ratio *β*-Ga_2_O_3_ nanorods was attributed to the oriented attachment mechanism that was present during the hydrothermal synthesis. A field-effect transistor was fabricated using the high-aspect ratio *β*-Ga_2_O_3_ nanorod, and it exhibited the typical charge transfer properties of an n-type semiconductor. This facile approach to forming high-aspect ratio nanorods without any surfactants or additives can broaden the science of *β*-Ga_2_O_3_ and expedite the integration of one-dimensional *β*-Ga_2_O_3_ into future electronics, sensors, and optoelectronics.

## 1. Introduction 

Gallium oxide in general and *β*-Ga_2_O_3_ in particular are getting more attention as exciting wide bandgap and nearly direct bandgap semiconductors (WBSes) [1,2,3,4]. The research and development around the Ga_2_O_3_-based semiconductor has offered substantial benefits to areas such as power electronics, high-speed electronics, photoelectrochemistry, photocatalysis, and gas/photon sensors [5,6]. Like other semiconductors, the low-dimensional properties of *β*-Ga_2_O_3_ have broadened the science of the materials, eventually leading to the promotion of innovative technologies, which have in turn promoted the development of new materials [7,8,9,10]. Numerous studies have been conducted on the synthesis and characterization of one-dimensional (1D) and two-dimensional (2D) *β*-Ga_2_O_3_ [11,12,13] mainly via chemical reaction-based approaches such as hydrolysis, sol–gel methods, electrospinning, and the hydrothermal method [14,15,16,17,18,19,20]. Among these various methods, *β*-Ga_2_O_3_ prepared via the hydrothermal method has been popular due to the resulting high crystallinity [21,22,23,24,25,26]. Furthermore, the hydrothermal method allows the formed *β*-Ga_2_O_3_ to have different faces of nanorods and/or nanoparticles, resulting in different surface area to volume ratios and/or aspect ratios. In fact, a minor change in aspect ratio can substantially change the optical, biological, and electrical properties. To elongate a certain desired direction, Ga_2_O_3_ nanorods are often synthesized via the catalytic chemical vapor deposition method and laser ablation, which requires elevated temperature reactions [27,28]. On the other hand, template-assisted and surfactant-assisted hydrothermal methods have also been developed to form Ga_2_O_3_ nanorods [28,29,30,31]. However, these methods bring up additional issues such as cross-contamination, unintentional doping, and increases in product costs [24,32]. In this study, a simple and facile method is introduced to form high-aspect ratio nanorods without any surfactants or additives, which can broaden the science of *β*-Ga_2_O_3_ and expedite the integration of 1D *β*-Ga_2_O_3_ into future electronics, sensors, and optoelectronics

## 2. Experimental

### 2.1. Precipitated *α*-GaOOH, Hydrothermal-Synthesized α-GaOOH, and β-Ga_2_O_3_ Nanorods

0.1 M Gallium(III) nitrate hydrate (Ga(NO_3_)_3_∙*x*H_2_O) was dissolved in 50 mL of deionized (DI) water via magnetic stirring at room temperature. The resultant solution was clear with a pH of 2.5. The solution was mixed with an ammonium hydroxide solution (from 28 to 30% NH_3_ in solution) on a hot plate at 60 °C to reach pH 10; the pH value changed in the range between 10.2 and 10.0 depending on the aging time. The supersaturated solution was subsequently aged from 10 min to 6 h in the beaker with magnetic stirring at 60 °C; there were no changes in process conditions other than the aging time. Next, at different aging times, the solution was transferred to a Teflon-lined stainless-steel autoclave that was sealed and heated in an electric oven at 140 °C for 10 h. (This method is known as hydrothermal synthesis). The autoclave was then naturally cooled down to room temperature. Finally, *α*-GaOOH was collected and washed several times with DI water to remove the residual reagents and dried in an oven at 70 °C for 6 h. The *α*-GaOOH nanorods turned into *β*-Ga_2_O_3_ nanorods after an annealing process at 1000 °C for 5 h. (This method is known as the calcination process). 

### 2.2. Material Characterization

The morphologies and sizes of the *α-*GaOOH and *β*-Ga_2_O_3_ nanorods at different aging times were characterized using a field-emission scanning electron microscope (FESEM, JSM-7100F, JEOL, Peabody, MA, USA). The structure and crystallinity of the nanorods were examined using powder X-ray diffraction (XRD, SMARTLAB, RIGAKU, Tokyo, Japan) with Cu K*α* radiation (λ = 0.154 nm) and high-resolution transmission electron microscopy (HRTEM, JEM 2100F, JEOL, Peabody, MA, USA; point resolution: 0.24 nm, lattice resolution: 0.1 nm) operating at an acceleration voltage of 200 kV. A Fourier transform infrared (FTIR, CARY670, Agilent, Santa Clara, CA, USA) spectrometer was also employed to analyze the chemical bonding of the *α-*GaOOH and *β*-Ga_2_O_3_ in the wavenumber range of 4000–450 cm^−1^. 

### 2.3. Fabrication of the Ga_2_O_3_ Nanorods Field-Effect Transistor (FET) 

The high-aspect ratios *β*-Ga_2_O_3_ nanorods were mechanically transferred onto the back-gated SiO_2_/p^+^ Si (300 nm/500 mm) substrate. The 10-μm spaced source/drain contacts (100 μm × 100 μm) were patterned on top of *β*-Ga_2_O_3_ nanorods using a conventional photolithography process followed by lift-off processes. Ti (5 nm) and TiN (500 nm) were deposited via physical vapor deposition for the metal contacts. Before electrical measurements, the devices underwent an annealing process at 300 °C for 3 h to improve their contact resistance. Electrical characterizations were performed using current-voltage measurements (Keithley 4200A-SCS, Tektronix, Beaverton, OR, USA) at room temperature.

## 3. Results and Discussion

**Figure 1:**
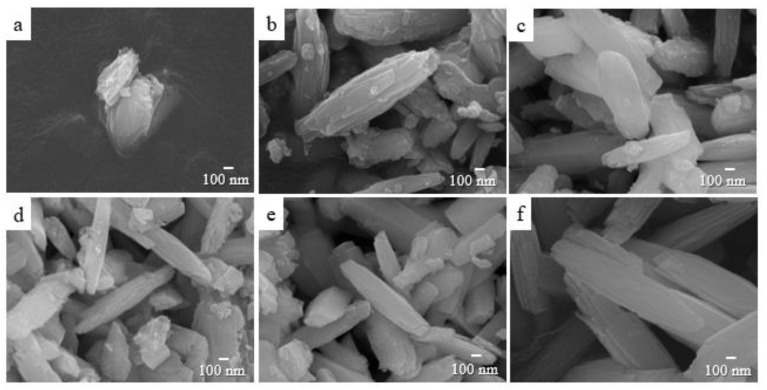
FESEM images of the precipitated amorphous nanostructure aged at 60 °C for (**a**) 10 min, and the precipitated *α*-GaOOH aged at 60 °C for (**b**) 1 h, (**c**) 1.5 h, (**d**) 2 h, (**e**) 3 h, and (**f**) 6 h.

Figure 1 shows the FESEM images of the precipitated nanostructures obtained from the supersaturated solution at different aging times. In the presence of alkali, the *α*-GaOOH nanoparticles precipitated immediately due to the reaction between the hydrated gallium as Ga(OH)_3_ and ammonium hydroxide in the supersaturated condition [33,34]. The unstable nanoparticles were subsequently aggregated by the well-known van der Waals force to form bigger nanoparticles [35]. Under this condition, the *α*-GaOOH nanoparticles selectively absorbed OH^−^ ions on all specific surfaces and formed a primary nanoplate along the preferential direction with a lower surface energy [30,33]. Aiding in lowering the surface energy, this primary nanoplate aggregated to form a stacked anisotropic nanoplatelet with a face-to-face plane that finally formed spindle-like *α*-GaOOH nanorods via the growth mechanism known as Ostwald ripening and oriented attachment [36]. The aggregated nanostructure at the aging time of 10 min (Figure 1a) turned out to be amorphous according to the XRD. As the aging time increased, aside from small nanoparticles, well-defined prismatic nanorods with specific facets (spindle-like nanorods) appeared at 1 h of aging, as shown in Figure 1b. The FESEM image showed that these spindle-like nanorods were composed of face-to-face stacked anisotropic nanoplatelets. In other words, the nanoplates, aggregating with the face-to-face plane to reduce the surface energy of the primary nanoplates, eventually formed spindle-like nanorods [33,37,38]. As the aging time increased further, the spindle-like nanorods dominated uniformly at the expense of the small nanoparticles, and eventually prism-like nanorods dominated without small nanoparticles after 6 h of aging. The above observation presumably suggested that the face-to-face stacked anisotropic nanoplatelets assembled the spindle-like nanorods.

**Figure 2:**
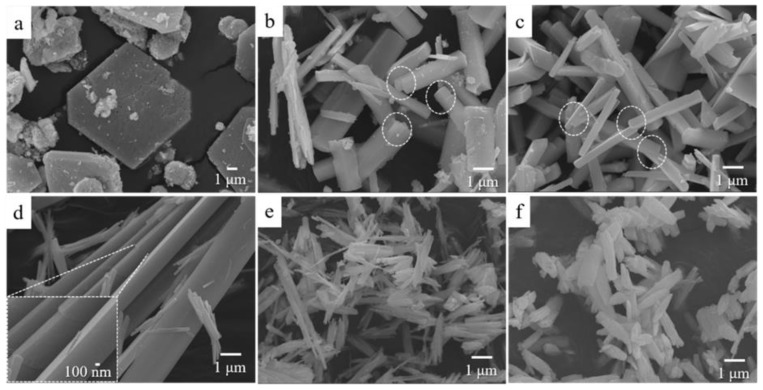
FESEM images of the *β*-Ga_2_O_3_ nanorods obtained from the hydrothermal synthesis at the aging times of (**a**) 10 min, (**b**) 1 h, (**c**) 1.5 h, (**d**) 2 h, (**e**) 3 h, and (**f**) 6 h, followed by calcination at 1000 °C for 5 h. The dotted circle indicates the area where the oriental attachment presumably happened.

Figure 2 shows the FESEM images of the various *β*-Ga_2_O_3_ nanorods that aged from 10 min to 6 h, followed by the identical hydrothermal and calcination processes. The FESEM images of the hydrothermal-synthesized *α*-GaOOH nanorods before the calcination process are not shown in this work because no notable differences were observed between the hydrothermal-synthesized *α*-GaOOH nanorods and calcinated *β*-Ga_2_O_3_ nanorods via FESEM. Nevertheless, the thermogravimetry (TGA) and differential scanning calorimeter (DSC) analysis revealed that this phase transformation was accompanied by a 2–8.8% weight loss [39,40]. For the 10-min aging condition, diverse morphologies of 3D flower-like and 2D sheet-like nanostructures as well as small nanoparticles were observed, as shown in Figure 2a, while nanorod structures appeared at aging times in the range from 1 h to 6 h, as shown in Figure 2b–f. A detailed discussion on these differences is outside the scope in this work; focus is centered on the formation of the high-aspect-ratio nanorods. Figure 2b showed that the products obtained after 1 h of aging followed by hydrothermal and calcination processes were comprised of prism-like nanorods and spindle-like nanorods as well as nanoparticles. As the aging time increased further, the prism-like nanorods with smooth surfaces (Figure 2b,c) turned into bundles of small nanorods with split structures (Figure 2e,f)). The edges of the split structure became grounded while the size of the nanorods remained unchanged at 6 h of aging, as shown in Figure 2f. It was interesting to notice that there was a transition condition where both the prism-like nanorods and nanorod bundles with split structures coexisted. The formed prism-like nanorods at this transition condition (Figure 2d) showed an extremely high aspect ratio without any surfactants or additives involved.

The comparison between Figure 1 and Figure 2 revealed that the lengths of the *β*-Ga_2_O_3_ nanorods (Figure 2) were longer than those of the precipitated *α*-GaOOH nanorods (Figure 1). Furthermore, the results showed that the morphology changes of the precipitated *α*-GaOOH nanorods depending on the different aging times (Figure 1) were insignificant, while the morphology changes of the hydrothermal-synthesized *α*-GaOOH nanorods (Figure 2) were significant. This implied that the precipitated products played a very important role for the diverse morphologies of the hydrothermal-synthesized products. 

**Figure 3:**
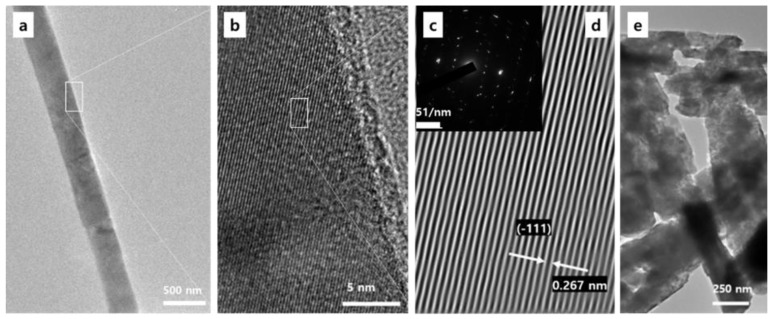
(**a**,**b**) TEM image of the high-aspect ratio *β*-Ga_2_O_3_ nanorods obtained from the hydrothermal synthesis at the aging times of 2 h followed by the calcination at 1000 °C for 5 h. (**c**) Corresponding electron diffraction pattern of the selected area. (**d**) The atomic-scale image from the Fourier transform of the the diffraction pattern in (**c**). (**e**) TEM image of the conventional *β*-Ga_2_O_3_ nanorods.

Figure 3a,b show the TEM images of the typical high-aspect ratio *β*-Ga_2_O_3_ nanorods. While the reported high-aspect ratio GaOOH was in tube (hollow cylinder) form [41], the high-aspect ratio *β*-Ga_2_O_3_ in this work was in nanorod form. The electron diffraction pattern of the selected area is shown in Figure 3c, and the atomic-scale image in Figure 3d is the Fourier transform of the the diffraction pattern. The results revealed that the formed high-aspect ratio *β*-Ga_2_O_3_ possessed excellent crystallinity with an interplanar spacing of about 0.267 nm, which corresponded to the ($\bar{1}$ 1 1-) crystal plane of monoclinic Ga_2_O_3_. Other than the high-aspect ratio *β*-Ga_2_O_3_, conventional *β*-Ga_2_O_3_ nanorods were also crystalized, as shown in Figure 3e.

**Figure 4:**
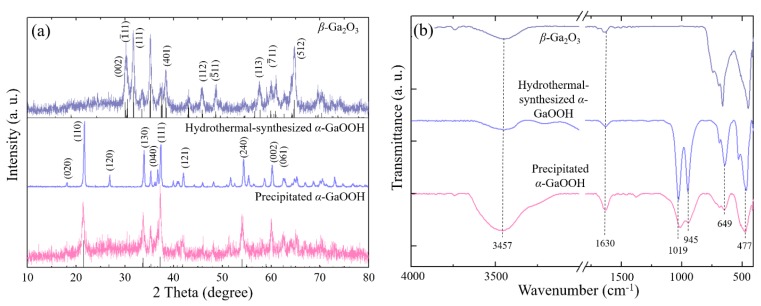
(**a**) XRD patterns and (**b**) FTIR spectra of the precipitated *α*-GaOOH nanorods with 2 h of aging, hydrothermal-synthesized *α*-GaOOH nanorods at 140 °C for 10 h, and *β*-Ga_2_O_3_ nanorods with 2 h of aging followed by the hydrothermal synthesis and calcination at 1000 °C for 5 h. It was noted that aside from the 2-h aging sample, 10-min, 1-h, 1.5-h, 2.5-h, 3-h, and 6-h samples were also examined using XRD and FTIR, but no noticeable changes were observed in the ranges from 1 h to 6 h.

The crystallinity of the obtained nanostructures was further characterized using an XRD analysis, as shown in Figure 4a. The X-ray diffraction patterns of the precipitated and the hydrothermal-synthesized *α*-GaOOH nanorod with 2 h of aging were perfectly indexed to the orthorhombic *α*-GaOOH phase with the following cell constants: *a* = 4.58 Å, *b* = 9.80 Å, *c* = 2.97 Å. This agrees well with the literature (JCPDS card No. 06-0180). In detail, the intensity of the hydrothermal-synthesized *α*-GaOOH nanorods was much sharper and stronger than that of the precipitated *α*-GaOOH nanorods. This indicated that the precipitated nanorods retained their orthorhombic *α*-GaOOH structures even after the hydrothermal process, and the hydrothermal process enhanced the crystal growth and the crystallinity of the *α*-GaOOH nanorods. The hydrothermal-synthesized *α*-GaOOH transformed into the monoclinic *β*-Ga_2_O_3_ nanorods (JCPDS card No. 76-0573) via the calcination at 1000 °C for 5 h, as shown in Figure 4a. It was noted that the 10-min aged products were found to be amorphous because no obvious diffraction peaks were observed via XRD. It took around 1 h of aging for the amorphous nanoparticles to aggregate with each other to form specific facets.

The chemical structures of the nanorods were also characterized using FTIR spectroscopy in the range from 4000 to 450 cm^−1^ regions. Figure 4b shows the chemical structures of the precipitated *α*-GaOOH nanorods, the hydrothermal-synthesized *α*-GaOOH nanorods, and *β*-Ga_2_O_3_ nanorods with 2 h of aging. The broad bands at around 3457 cm^−1^ and 1630 cm^−1^ from the precipitated *α*-GaOOH nanorods with 2 h of aging represented the stretching vibration of the H–O–H and O–H bonds, respectively [42,43]. This indicated that water molecules remained in the precipitated *α*-GaOOH nanorods [44]. Other than the water molecule-related peaks, the strong bending vibrations at 1019 and 945 cm^−1^ were attributed to the Ga–OH bands in the *α*-GaOOH nanorods [26,34]. The strong bands at 649 cm^−1^ and 477 cm^−1^ represented the Ga–O bending vibration and Ga–O stretching vibration [45], respectively. After the hydrothermal and further calcination process, the peaks representing the Ga-related peaks enhanced while the water molecule-related peaks diminished.

**Figure 5:**
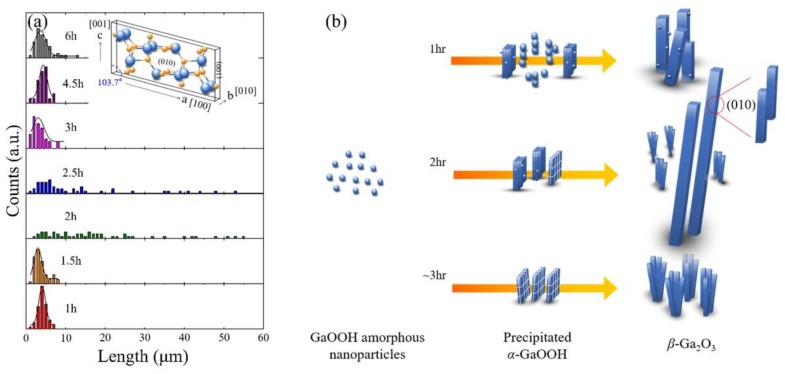
(**a**) Histogram of the size distribution and (**b**) proposed growth mechanism of *β*-Ga_2_O_3_ nanorods depending at different aging times. Under the insufficient aging time (below 2 h in these experimental conditions), the uncompleted nanoparticles prevented the attachment of the spindle-like nanorods parallel to the plane, causing a prism-like nanord with nanoparticles. On the other hand, under the excessive aging time (above 3 h in these experimental conditons), the precipitated nanorods with smooth surfaces were continually and repeatedly dissolved and re-assembled in the supersaturated solution. The re-assembled nanorods at longer aging times presumably possessed more defects and mismatches, which also prevented the attachment of nanorods parallel to the plane and caused the crystal to split, leading to bundles of small nanorods of split structures.

A length distribution histogram of the *β*-Ga_2_O_3_ nanorods obtained at different aging times is shown in Figure 5a, which was extracted from the SEM images by considering 100 nanostructures. The result revealed two key features: (i) regardless of the different aging times (except 2 and 2.5 h of aging), the mean length of the final *β*-Ga_2_O_3_ nanorods remained around 3 μm with a gaussian normal distribution; (ii) on the contrary, the size distribution of the *β*-Ga_2_O_3_ nanorods at 2 h and 2.5 h of aging became uneven. This indicated that chemical reactions such as nucleation, growth and dissolution, and re-growth et al. played a significant role for all conditions other than 2 and 2.5 h of aging, because these times showed gaussian normal distributions. Unlike the gaussian normal distribution, the final *β*-Ga_2_O_3_ nanorods at 2 h and 2.5 h of aging showed uneven size distributions. This indicated that aside from the normal chemical reaction, other mechanisms such as oriental attachment were presumably a main driving force in forming the high-aspect ratio *β*-Ga_2_O_3_ nanorods.

It is worthwhile postulating on the growth mechanism of the high-aspect ratio *β*-Ga_2_O_3_ nanorods. It has been reported that the morphology and size of hydrothermal-synthesized *α*-GaOOH or *β*-Ga_2_O_3_ nanorods can be affected by pH values, hydrothermal synthesis times/temperatures, reaction temperatures, and alkali chemicals [33,34,41,46,47]. We argue that aside from these factors, the aging time used to form the precipitated products in the supersaturated solution could also significantly affect the morphology and size of the *α*-GaOOH or *β*-Ga_2_O_3_ nanorods. The SEM images in Figure 2b,c revealed that the nanorods with prism-like crystals tended to attach in certain planes because they reduced the total surface energy of the nanoparticles in the solution [47,48]. As the aging time increased, this preferred attachment parallel to the plane could be enhanced, thereby forming high-aspect ratio nanorods, as shown in Figure 2d. The attachment of the nanorods parallel to the plane could be supported via the high-resolution SEM image (inset) in Figure 2d. Based on the hypothesis, the re-attachment in the plane was preferable at 2 h of aging, which eventually caused the high-aspect ratio *β*-Ga_2_O_3_ nanorods with prism-like crystals with an uneven distribution. It was noted that the precipitated nanorods with smooth surfaces in Figure 1 were dissolved and re-assembled continually in the supersaturated solution. Unlike the newly-formed nanorods at shorter aging times, the re-assembled nanorods at longer aging times presumably possessed more defects and mismatches. These accumulated defects and mismatches on surface of the re-assembled nanorods prevented the attachment of nanorods parallel to the plane, causing the crystal to split and leading to bundles of small nanorods of split structures. The overall mechanism is described in Figure 5b. 

**Figure 6:**
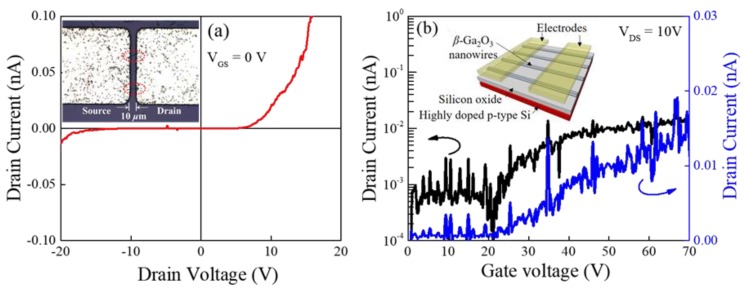
(**a**) output (I_DS_-V_DS_) and (**b**) transfer (I_DS_-V_GS_) characteristics of the Ga_2_O_3_ nanorod FETs. Inset image of (**a**): optical microscope image of *β*-Ga_2_O_3_ FET (source-drain distance: 10 μm). Inset image of (**b**): a schematic drawing of the bird’s eye view of the Ga_2_O_3_ FET. This limited current density the Ga_2_O_3_ nanorod FETs was comparable to the semi-insulating Ga_2_O_3_ bulk FETs without source and drain doping [51].

The formed high-aspect ratio *β*-Ga_2_O_3_ nanorods via hydrothermal synthesis followed by the calcination process were transferred into the back-gated SiO_2_/p^+^ Si substrate, and then, a conventional lift-off process was performed to fabricate the device, as shown in the inset of Figure 6. Figure 6a,b shows output (I_DS_-V_DS_) and transfer (I_DS_-V_GS_) characteristics of the Ga_2_O_3_ nanorod FETs. Figure 6a showed that at least 10 V was required to overcome the Schottky barrier at the edge of the source-to-channel region. Then the V_DS_ = 10 V was applied to evaluate the transfer curve as shown in Figure 6b. It reveals the typical charge transfer properties of an n-type semiconductor. The gate modulation was not impressive, mainly due to the low on-state current limited by the high contact resistance, which would be improved by adopting a doping technique at the source/drain and channel regions. It was reported that this unintentionally n-type behavior in the undoped *β*-Ga_2_O_3_ was attributed to the the oxygen deficiency and/or residual impurities [49,50].

## 4. Conclusions

High-aspect ratio *β*-Ga_2_O_3_ nanorods were formed using gallium oxyhydroxide and ammonia hydroxide via a hydrothermal synthesis followed by a subsequent calcination process. The results showed that the morphology changes of the precipitated *α*-GaOOH nanorods depending on the different aging times were insignificant, while those of the hydrothermal-synthesized *α*-GaOOH nanorods were significant. This implied that the precipitated products played a very important role in the final products after the hydrothermal process. The formation of the high-aspect ratio *β*-Ga_2_O_3_ nanorods was presumably attributed to the re-attachment of the newly formed nanorodes in the plane of the nanostructures. Furthermore, a field-effect transistor was fabricated using the *β*-Ga_2_O_3_ nanorod, showing the typical charge transfer properties of an n-type semiconductor. This facile approach to forming a high-aspect ratio nanorods without any surfactants or additives can broaden *β*-Ga_2_O_3_ science and expedite the integration of one-dimensional *β*-Ga_2_O_3_ into future electronics, sensors, and optoelectronics.
